# Epilepsy in patients with congenital heart disease: A nationwide cohort study

**DOI:** 10.1002/brb3.2699

**Published:** 2022-07-08

**Authors:** Johan Zelano, Mikael Dellborg, Peter Eriksson, Zacharias Mandalenakis

**Affiliations:** ^1^ Department of Neuroscience and Physiology Sahlgrenska Academy, University of Gothenburg Gothenburg Sweden; ^2^ Wallenberg Center of Molecular and Translational Medicine Sahlgrenska Academy, University of Gothenburg Gothenburg Sweden; ^3^ Department of Neurology Sahlgrenska University Hospital Gothenburg Sweden; ^4^ Institute of Medicine, Department of Molecular and Clinical Medicine Sahlgrenska Academy, University of Gothenburg Gothenburg Sweden; ^5^ Adult Congenital Heart Disease Center, Department of Medicine Sahlgrenska Academy, University of Gothenburg Gothenburg Sweden

**Keywords:** congenital heart disease, epidemiology, epilepsy

## Abstract

**Background:**

Congenital heart disease (CHD) is the most common congenital defect, and reports suggest an increased risk of subsequent epilepsy. We used Swedish comprehensive population‐based registers to investigate the risk of epilepsy in patients with CHD compared to matched controls and identify underlying factors of epilepsy.

**Methods:**

All patients with CHD born between 1970 and 2017 and 10 age‐ and sex‐matched controls were included. Epilepsy was ascertained by International Statistical Classification of Diseases and Related Health Problems codes, and the cumulative hazard of epilepsy was described using Cox regression.

**Results:**

The study cohort consisted of 71,941 patients with CHD and 714,462 matched controls. The cumulative incidence of epilepsy in the study period was 3% in patients with CHD and 0.9% in controls. The risk of epilepsy was 3.6 times higher (95%, confidence interval: 3.4–3.8) in patients with CHD than in controls. Among patients with CHD, several brain comorbidities, including intellectual disability and stroke, as well as having undergone more than two cardiac interventions were significantly associated with epilepsy in a multivariable model.

**Conclusions:**

In this nationwide, register‐based cohort study, we found an almost fourfold increased risk of epilepsy in patients with CHD compared to controls; however, the absolute risk was low. Among the identified risk factors, stroke may be potentially preventable.

## Graphical abstract

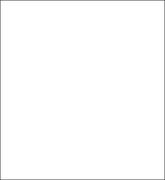

## 1 INTRODUCTION

Congenital heart disease (CHD) is the most common congenital defect and occurs in 1% of live births (Khoshnood et al., 2012). The survival of children with CHD has increased exponentially over the last decades, and today, more than 97% will reach adulthood (Mandalenakis et al., 2020, 2017; Moons et al., 2010). With increased survival, patients with CHD face the risk of developing acquired diseases and long‐term complications such as epilepsy. Previous studies have described an elevated risk of epilepsy in patients with CHD. Surgery itself carries risks—in 10 children with seizures in the month surrounding their cardiac surgery, reasons included stroke or procedurally related hypoxic‐ischemic injury (Desnous et al., 2019). A nationwide study from Denmark reported a 5% incidence of epilepsy in patients diagnosed with CHD before the age of 15 years, and a risk of epilepsy related to the number of cardiac procedures (Leisner et al., 2016). Stroke is also a risk factor for subsequent epilepsy in infants undergoing cardiac surgery for CHD (Ghosh et al., 2020).

The increased risk of epilepsy in the CHD population raises several questions. Could associated congenital syndromes confound the analyses? Does CHD itself, without cardiac surgery, also increase the risk of stroke and therefore epilepsy?

We used Swedish comprehensive population‐based registers to study the risk of epilepsy in patients with CHD compared to matched controls, with the aim of identifying risk factors furthering pathophysiological understanding.

## 2 METHODS

### 2.1 Study population

Data from the Swedish National Patient Register (inpatient, outpatient, and Cause of Death register) were linked to identify patients with a diagnosis of CHD. In the present study, we included all patients with CHD who were born between January 1, 1970 and December 31, 2017. Ten random individuals from the Total Population Register were selected with matched birth year and sex for each CHD patient. The study population and design have been described in detail elsewhere (Mandalenakis et al., 2020, 2017, 2018). The randomly selected persons from the Total Population Register (“controls”) are representative of the general population cohort. Follow‐up and comorbidity variables were included until December 31, 2017 or death.

### 2.2 Definitions

Diagnoses were identified according to the International Statistical Classification of Diseases and Related Health Problems (ICD). CHD, epilepsy, epilepsy‐related codes, head trauma, brain tumors, intellectual disability, Down's syndrome, alcohol or substance abuse ICH, ischemic stroke, and prematurity/low‐birth weight were defined as the occurrence of at least one relevant code (Table 1).

The CHD population was grouped into six different lesion groups according to a hierarchical classification system (Botto) based on the severity of the defect (Botto et al., 2007; Liu et al., 2016). The Botto classification is hierarchical: Lesion group 1 was defined as patients with conotruncal defects (such as common arterial trunk, transposition of the great vessels, double‐outlet right ventricle, double‐outlet left ventricle, discordant atrioventricular connection, tetralogy of Fallot, and aortopulmonary septal defect). Lesion group 2 was defined as patients with nonconotruncal defects (such as endocardial cushion defects, common ventricle, and hypoplastic left heart syndrome). Lesion group 3 was defined as patients with coarctation of the aorta. Lesion group 4 was defined as patients with ventricular septal defects. Lesion group 5 was defined as patients with atrial septal defects. Lesion group 6 included all other heart and circulatory system anomalies, and all other CHD diagnoses not included in lesion groups 1−5. Cardiac intervention was defined as congenital cardiac surgery or congenital cardiac interventional catheterization according to the classification of operations or classification of procedures (The National Board of Health & Welfare, 1993, 1997).

### 2.3 Statistical analysis

The cumulative incidence was calculated as the proportion of individuals with a diagnosis of epilepsy at any time during the study period. Confidence intervals (CIs) of proportions were calculated by the modified Wald method. In Kaplan–Meier and Cox proportional hazard analyses, participants were censored on December 31, 2017 or death or an event defined as the occurrence of epilepsy. Sensitivity analyses were performed without individuals with intellectual disability or prematurity to ensure that the entire association between epilepsy and CHD did not result from these conditions. A separate sensitivity analysis included only individuals with at least 2 years of follow‐up. In the multivariate Cox regression, stepwise forward selection was used, which allowed effects already in the model to be removed at each step. Comorbidities were time‐updated covariates with the first occurrence of a diagnostic code as occurrence. Statistical analyses were performed in STATA by Statistiska konsultgruppen (commercial, Gothenburg, Sweden).

### 2.4 Ethical permission

The present study was approved by the Gothenburg Regional Research Ethics Board (Gpg 912–16, T 616–18) and accompanied the ethical guidelines of the Declaration of Helsinki.

## 3 RESULTS

The study cohort consisted of 71,941 patients with CHD and 714,462 matched controls (Table 1). A greater proportion of patients with CHD (8%) died before the end of the observation period compared to their controls (0.6%). Intellectual disability, stroke, and prematurity were more common among cases. Twenty‐five percent of patients with CHD had undergone cardiac intervention.

**TABLE 1:** Demographics and comorbidities of the study cohort, stratified by exposure (congenital heart disease [CHD] or control) and presence of epilepsy

Variable	CHD (*n* = 71,941)	CHD with epilepsy(*n* = 2136)	Controls(*n* = 714,462)	Controls with epilepsy(*n* = 6222)
Dead	5768 (8.0%)	263 (12.3%)	4484 (0.6%)	252 (4.1%)
Age (at the end of follow‐up)				
0−10	30,451 (42.3%)	571 (26.7%)	260,283 (36.4%)	1273 (20.5%)
11–20	17,779 (24.7%)	579 (27.1%)	180,187 (25.2%)	1873 (30.1%)
21–30	12,525 (17.4%)	530 (24.8%)	138,818 (19.4%)	1621 (26.1%)
31–40	6988 (9.7%)	292 (13.7%)	82,870 (11.6%)	903 (14.5%)
41–50	4198 (5.8%)	164 (7.7%)	52,304 (7.3%)	552 (8.9%)
Sex				
Male	36,102 (50.2%)	1099 (51.5%)	361,020 (50.5%)	3199 (51.4%)
Female	35,839 (49.8%)	1037 (48.5%)	353,442 (49.5%)	3023 (48.6%)
Comorbidities				
Head trauma	2242 (3.1%)	188 (8.8%)	16,500 (2.3%)	478 (7.7%)
Tumors	6434 (8.9%)	314 (14.7%)	50,268 (7.0%)	852 (13.7%)
Brain tumors	128 (0.2%)	41 (1.9%)	728 (0.1%)	161 (2.6%)
Intellectual disability	3408 (4.7%)	864 (40.4%)	5056 (0.7%)	1169 (18.8%)
Down's syndrome	3152 (4.4%)	201 (9.4%)	298 (0.0%)	16 (0.3%)
Substance abuse	486 (0.7%)	40 (1.9%)	3552 (0.5%)	199 (3.2%)
ICH	246 (0.3%)	59 (2.8%)	408 (0.1%)	72 (1.2%)
Brain Infarction	999 (1.4%)	139 (6.5%)	550 (0.1%)	97 (1.6%)
Prematurity	8272 (11.5%)	401 (18.8%)	1782 (0.2%)	98 (1.6%)
CHD‐related procedures				
Cardiac intervention	18,263 (25.4%)	690 (32.3%)	148 (0.0%)	10 (0.2%)
Number of cardiac interventions				
0	53,678 (74.6%)	1446 (67.7%)	714,314 (100.0%)	6212 (99.8%)
1	14,267 (19.8%)	521 (24.4%)	131 (0.0%)	8 (0.1%)
2	2364 (3.3%)	97 (4.5%)	14 (0.0%)	2 (0.0%)
>2	1632 (2.3%)	72 (3.4%)	3 (0.0%)	0 (0.0%)

Abbreviation: ICH, intracerebral hemorrhage.

### 3.1 Incidence of epilepsy

**FIGURE 1:**
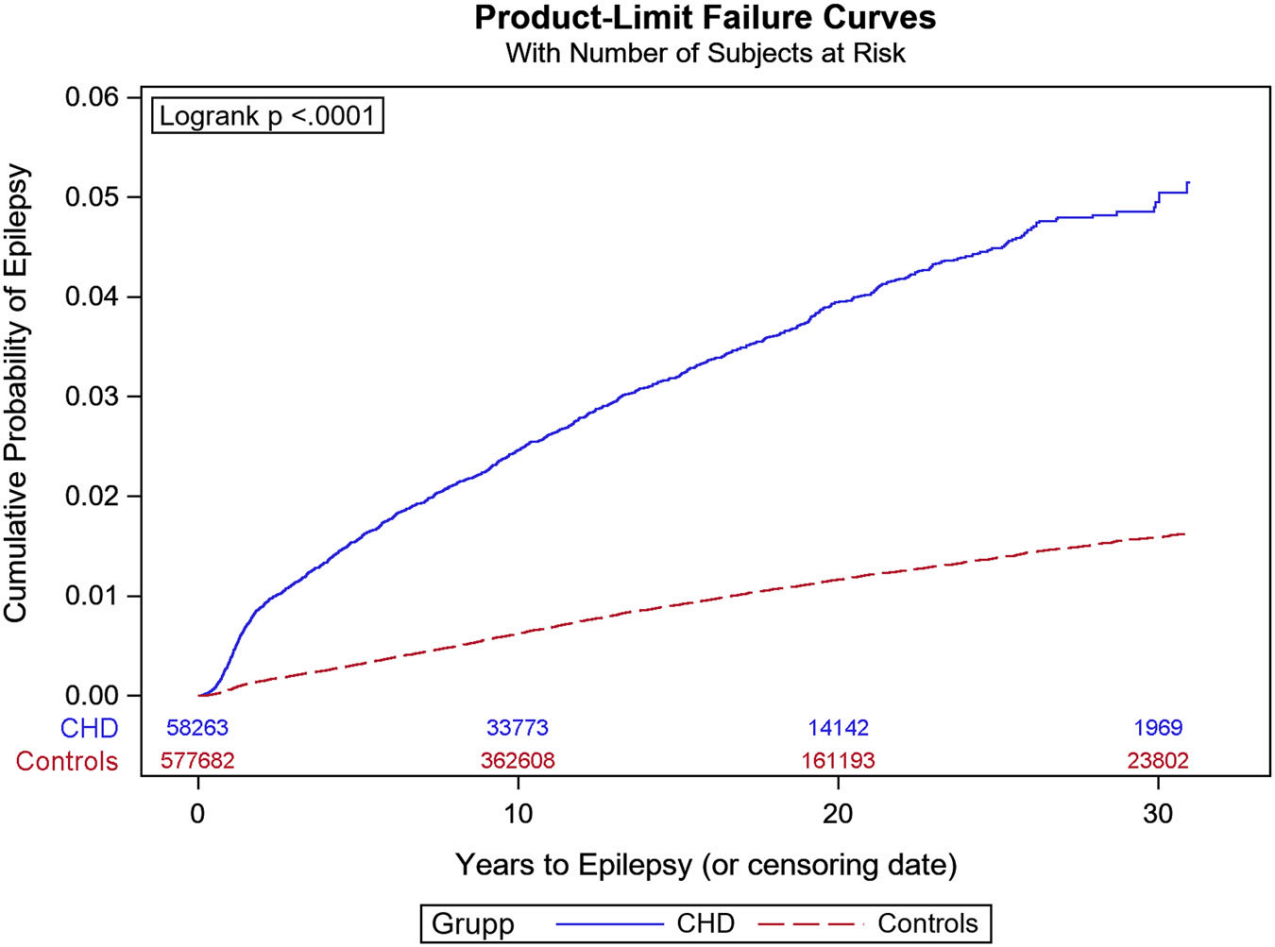
Kaplan–Meier risk of epilepsy in patients with congenital heart disease (CHD) and controls. The numbers above years indicate persons at risk

**FIGURE 2:**
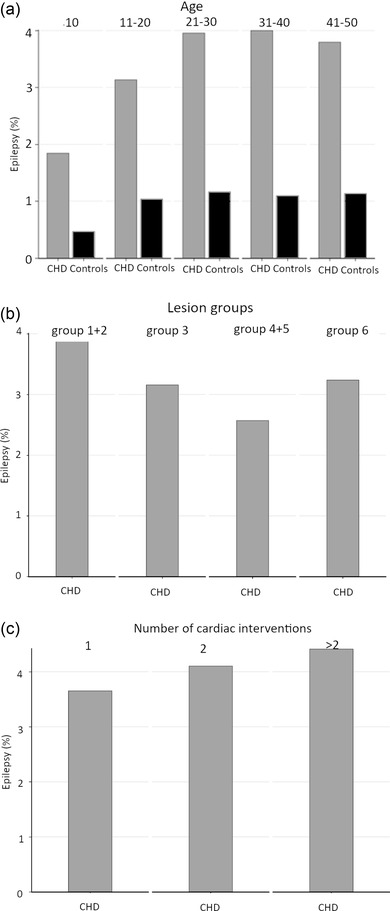
Cumulative incidence of epilepsy during the study period in different participant strata. (a) By age at follow‐up, (b) by congenital heart disease (CHD) severity as categorized by lesion groups, and (c) by number of cardiac interventions

The cumulative incidence of epilepsy during the study period was 3.0% (95%, CI: 2.9‐3.1) in cases and 0.9% (95%, CI: 0.9–0.9) in controls (Table 2). The cumulative incidence of any epilepsy, seizure, or status epilepticus was 4.0% (95%, CI: 3.9–4.2) in cases and 1.1% (95%, CI: 1.1–1.1) in controls. In Kaplan–Meier analysis, the survival‐adjusted risk was 5% at 30 years, and separation between patients with CHD and controls continued to grow throughout the study period (Figure 1). The cumulative incidence of epilepsy for individuals with optimal coverage of national registers, born 1980–2010, was 3.3% (95%, CI: 3.2–3.5). The cumulative incidence of epilepsy increased with age at the end of follow‐up until age 21 (Figure 2a). The highest risk of epilepsy was seen in the most complex CHD, such as lesion groups 1–2 (Figure 2b) and in patients undergoing > 2 cardiac interventions (Figure 2c). In Cox regression, the HR of epilepsy in individuals with CHD compared to controls was 3.6 (95%, CI: 3.4–3.9). In a multivariable model comprising age, sex and comorbidities, the HR for epilepsy in CHD was 2.5 (95%, CI: 2.4–2.7).

**TABLE 2:** Epilepsy‐related diagnoses in congenital heart disease (CHD) patients and controls. The frequencies and percentages indicate the presence of a code for epilepsy‐related conditions in exposed and unexposed members of the cohort

	All	
Variable	CHD (*n* = 71,941)	Controls (*n* = 714,462)
Epilepsy	2136 (3.0%)	6222 (0.9%)
Seizure	1355 (1.9%)	2414 (0.3%)
Status epilepticus	141 (0.2%)	295 (0.0%)
Any of the above	2894 (4.0%)	7823 (1.1%)

### 3.2 Risk factors among patients with congenital heart disease

Among patients with CHD, factors such as younger age, brain comorbidities, prematurity, substance abuse, and cardiac interventions were associated with an increased risk of epilepsy (Table 3). All brain comorbidities and having undergone more than two cardiac interventions remained significantly associated with epilepsy in a multivariable model.

**TABLE 3:** Cox regression of HR for epilepsy depending on different risk factors in patients with congenital heart disease (CHD)

	Univariate			Multivariate		
	HR	95% CI	*p*	HR	95% CI	*p*
Year of birth	1.013	1.01–1.02	.0005	1.015	1.01–1.02	<.0001
Male sex	1.069	0.97–1.18	.1835			
Comorbidities						
Head trauma	1.985	1.44–2.74	<.0001	1.643	1.19–2.27	.0026
Brain tumors	16.95	9.09–31.6	<.0001	11.79	6.28–22.1	<.0001
Intellectual disability/Down's syndrome	5.009	4.45–5.64	<.0001	4.747	4.21–5.35	<.0001
Substance abuse	7.168	3.34–15.4	<.0001	5.444	2.55–11.6	<.0001
ICH	13.50	8.68–21.0	<.0001	2.378	1.41–4.02	.0012
Ischemic stroke	14.80	10.9–20.0	<.0001	9.782	6.84–14.0	<.0001
Premature/low birth weight	1.955	1.74–2.20	<.0001	1.880	1.67–2.12	<.0001
CHD procedure						
Cardiac intervention	1.402	1.25–1.58	<.0001			
One CI	1.405	1.25–1.58	<.0001			
Two CIs	1.396	1.12–1.74	.0033			
More than two CIs	1.866	1.37–2.54	<.0001	1.672	1.23–2.28	.0012

Abbreviations: CI, cardiac intervention; ICH, intracerebral hemorrhage.

### 3.3 Sensitivity analyses

Since CHD is associated with congenital syndromes, which may confound the risk of epilepsy, we excluded intellectual disability (a surrogate marker of such syndromes) and Down's syndrome in a sensitivity analysis. The HR of epilepsy remained higher in persons with CHD (2.6, 95%, CI: 2.4–2.8). Similarly, prematurity may be associated with both CHD and epilepsy, but if persons with prematurity were excluded, the HR of epilepsy in patients with CHD remained elevated at 3.3 (95% CI: 3.1–3.5). In another sensitivity analysis, we included only individuals with at least 2 years before censoring, which gave very similar uni‐ and multivariate HRs for risk factors among patients with CHD as the main analysis.

## 4 DISCUSSION

In the present nationwide, register‐based cohort study, we found a fourfold increased risk of developing epilepsy in patients with CHD compared with matched controls without CHD. We also identified a number of risk factors for subsequent epilepsy among patients with CHD. The number of cardiac interventions was associated with subsequent epilepsy.

The underlying mechanisms of the link between CHD and epilepsy have thus far not been extensively studied. We used nationwide registers to search for risk factors and identified several associations that can deepen the understanding of how CHD is linked to epilepsy. One possible explanation could be confounding by congenital syndromes, which could increase the risk of both epilepsy and CHD. However, in a multivariable model adjusting for intellectual disability and low birth weight, the risk of epilepsy associated with CHD remained significantly elevated. If patients with intellectual disability were excluded, CHD conferred an HR of epilepsy of 2.6. Similarly, if individuals with prematurity were excluded, the HR of epilepsy in patients with CHD was 3.3. Taken together, confounding by syndromes giving CHD as well as prematurity or intellectual disability does not seem to account for the entire risk increase associated with CHD. Stroke, another risk factor in cases with CHD, could be an intermediate variable in the CHD‐epilepsy link; it greatly increased the risk of epilepsy in our cohort, and several smaller studies have described plausible mechanistic links between CHD, stroke, and seizures related to cardiac interventions (Desnous et al., 2019; Ghosh et al., 2020). Having undergone cardiac intervention also carried an increased risk.

Our study is register‐based, with associated strengths and limitations. The detection of epilepsy should be sufficient—the positive predictive value of an ICD code for epilepsy in the NPR is 90% (Sveinsson et al., 2017). Although reporting to Swedish national registers is mandatory, all comorbidities have not been validated. Some risk factors are likely to be vulnerable to detection bias—it is, for instance, very likely that substance abuse is better detected in registers for patients with CHD than controls simply because of the more frequent interactions with the health care system in the former group. It is also possible that epilepsy could have been detected somewhat earlier in the CHD group than in the control group because of more frequent hospital contacts. For other comorbidities, such as brain tumors and stroke, this should be a smaller problem. Our overall risk estimate for epilepsy is in excellent agreement with a previous nationwide report from Denmark (Leisner et al., 2016).

A strength of our investigation is the long follow‐up allowed by comprehensive national registers, but this also adds to the complexity in interpretation of the findings. Treatment and diagnosis of CHD improved markedly during the course of our study, and epilepsy diagnostic criteria have been revised. Similarly, the diagnosis of and survival after several important variables, such as stroke, have improved, which increases the risk of epilepsy. More than 50% of CHDs are diagnosed within the first month of life in quality registry data, which also includes historical patients. Currently, almost all patients with complex CHD are diagnosed antenatal or before discharge. To account for shifts in diagnostic sensitivity and criteria, we included birth year as a covariate in our Cox analysis. Being born more recently was associated with an increased risk of epilepsy, which presumably does not represent a biological difference but rather temporal improvements in coverage of epilepsy and certain covariates in the NPR. Cohort effects should have been statistically accounted for by matching on birth year.

In summary, the association between CHD and epilepsy seems multifactorial, but our results suggest that some risk factors may be modifiable. More studies on the prevention of epilepsy in persons with CHD are motivated, for instance, studies on preventing and mitigating stroke. Another interesting issue is whether the timing of intervention for CHD has any influence on CHD risk.

## CONFLICT OF INTEREST

Dr. Zelano reports speaker honoraria for unbranded educations from Eisai and UCB and being an investigator in clinical trials sponsored by GW Pharma, UCB, Bial, and SK Life Science as an employee of Sahlgrenska University Hospital (no personal compensation). No other authors report any disclosures.

### PEER REVIEW

The peer review history for this article is available at https://publons.com/publon/10.1002/brb3.2699

## Supporting information

Supplemental table 1. ICD codes. SE = status epilepticus, ICH = intracerebral hemorrhage

## Data Availability

The data originate from nationwide registers available for research, but the data compiled for this study are protected by Swedish privacy laws and therefore are not available to other researchers.
