# Supplementary material for: Epilepsy in patients with congenital heart disease: A nationwide cohort study
Source: Brain Behav. 2022 Jul 8;12(8):e2699. doi: 10.1002/brb3.2699 (PMC9392522; doi:10.1002/brb3.2699)
Supplement: Supplementary file 1 — Supplemental table 1. ICD codes. SE = status epilepticus, ICH = intracerebral hemorrhage [file BRB3-12-e2699-s001.docx]

| **Condition** | **ICD-10** | **ICD-9** | **ICD-8** |
| --- | --- | --- | --- |
| CHD | Q20-26 | 745-747 | 746-747 |
| Epilepsy | G40 | 345 (not 345Q = SE) | 345 (not 342.20 = SE) |
| Epilepsy-related | G40, R568 (seizure), G41 (SE) | 345, 780D (seizure) | 345, 720.20 (seizure) |
| Head trauma | S00-06 | 850-854, 800-804 | 850-854, 800-804 |
| Brain tumours | C71, D42 | 191, 225 | 191, 225.00, 225.20 |
| Intellectual disability | F70-79 | 317–319 | 310-315 |
| Down’s syndrome | Q90 | 758A | 315.50-315.51 |
| Alcohol or substance abuse | F11-F19 | 303-304, 305X | 303-304 |
| ICH | I60-62 | 430-431 | 430-431 |
| Ischemic stroke | I63, I69 (if no I60-62) | 432-436, 438 | 432-436, 438 |
| Prematurity/low-birth weight | P07 | 764-765 | 777 |

Supplemental table 1. ICD-codes. SE=status epilepticus, ICH =intracerebral haemorhage
